# Evaluation of biological mechanisms of artemisinin on bovine mammary epithelial cells by integration of network pharmacology and TMT-based quantitative proteomics

**DOI:** 10.3389/fphar.2022.968149

**Published:** 2022-09-09

**Authors:** Jinjin Tong, Yang Sun, Ziyue Wang, Defeng Cui, Linshu Jiang

**Affiliations:** Beijing Key Laboratory for Dairy Cow Nutrition, Beijing University of Agriculture, Beijing, China

**Keywords:** artemisinin, network pharmacology, proteomics, dairy cow, bovine mammary

## Abstract

The sesquiterpene lactone, artemisinin, is a primary component of the medicinal plant *Artemisia annua* L., which has anti-inflammatory, antibacterial and antioxidant activities. However, the potential effects of artemisinin on the mammary gland of dairy cows and the underlying molecular mechanisms remain unclear. Here, we utilized systematic network pharmacology and proteomics to elucidate the mechanism by which artemisinin affects milk production and the proliferation of bovine mammary epithelial cells (BMECs). Nineteen bioactive compounds and 56 key targets were identified through database mining. To delineate the mechanism of artemisia’s activity, a protein-protein interaction network and integrated visual display were generated from bioinformatics assays to explore the relationships and interactions among the bioactive molecules and their targets. The gene ontology (GO) terms and kyoto encyclopedia of genes and genomes annotation suggested that the apoptotic process, cell division, p53 pathway, prolactin and PI3K-Akt pathways played vital roles in mammary gland development. Using proteomics analysis, we identified 122 up-regulated and 96 down-regulated differentially significant expressed proteins (DSEPs). The differentially significant expressed proteins had multiple biological functions associated with cell division, apoptosis, differentiation, and migration. Gene ontology enrichment analysis suggested that differentially significant expressed proteins may promote cell proliferation and regulate apoptosis in bovine mammary epithelial cells. Kyoto encyclopedia of genes and genomes pathway analysis indicated that several biological pathways, such as those involved in antigen processing and presentation, cell adhesion molecules and ribosomes, played significant roles in the effects of artemisinin on bovine mammary epithelial cells. These findings contribute to a comprehensive understanding of the mechanism by which artemisinin affects bovine mammary epithelial cells to improve mammary gland turnover by inducing cell proliferation and mammary gland development.

## Graphical Abstract

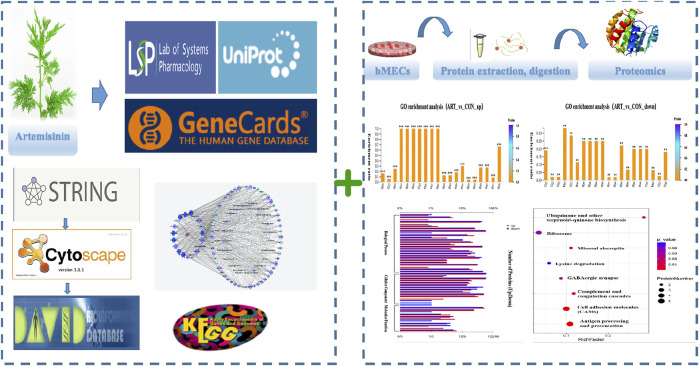

## 1 Introduction

Throughout their lifetimes, the mammary glands of dairy cows undergo periodic alterations associated with pregnancy, which include bovine mammary epithelial cell (BMEC) proliferation and differentiation, milk protein synthesis and secretion, and apoptosis (Turner and Huynh, 1991). Milk synthesis is one of the most important biological functions of the bovine mammary gland, which comprises a branching network of ducts that end with alveoli surrounding the lumen. The milk-producing ability of dairy cows is determined by these alveolar BMECs and depends on their number, activity, and secretory function (Yan Q. et al., 2019). Milk proteins and 50% of the fatty acids (FAs) in cow’s milk are produced within BMECs (McManaman, 2012). Therefore, f; ly6.

Artemisinin (ART) is a 1,2-trioxane drug extracted from *Artemisia annua* L (Paddon et al., 2013). ART has been reported to have anti-bacterial, anti-fungal, antioxidant, anti-tumor, and anti-inflammatory activities (Efferth, 2007; Konkimalla et al., 2008; Ferreira et al., 2010). Essential oil from *A. annua* inhibited *Staphylococcus aureus*, which is one of the causes of bovine subclinical mastitis, in a dose- and time-dependent manner *in vitro* (Bilia et al., 2014). Furthermore, incubation of LPS-activated neutrophils with extracts of *A. annua* significantly inhibited TNF-alpha production and reduced the inflammatory response in a dose-dependent manner (Sheena et al., 2015). Previous investigations indicated that *A. annua* effectively regulated the immune function and facilitated apoptosis in hepatoma cells (Yan L. et al., 2019). Interestingly, we found that feeding artemisinin extract to dairy cows with subclinical mastitis could significantly reduce the somatic cell count and improve milk quality, as well as increase milk production and antioxidant levels in mid-lactation dairy cows (Hou et al., 2019, 2020). However, the effects of artemisinin on the mammary gland are not clearly understood, particularly the molecular mechanisms underlying the effects of artemisinin on mammary epithelial cells that regulate milk biosynthesis.

The new discipline of network pharmacology (NP) is considered to be a useful technique, which integrates multi-omics to explore the activities and mechanisms of traditional Chinese medicine (TCM) formulations (Tian et al., 2019; Zhang et al., 2020). It provides a novel strategy for evaluating the multiple ingredients and targets of traditional Chinese medicines and identifying the primary active molecular compounds with therapeutic roles (Song et al., 2021). NP’s methods are different from the conventional one-target/one-drug research strategy as they take into account the complex interactions inherent in living systems between therapeutic agents and the pathophysiology involved in disease from a holistic standpoint (Bgab et al., 2021). Furthermore, proteome analyses based on mass spectrometry (MS) methods have become the go-to source for gathering data about the relative amounts of proteins and their changes. High-resolution, high-throughput MS is routinely used for identification and quantification of protein profiles in various tissues and cells for pinpointing novel targets (Wilhelm et al., 2014). Tandem mass tags (TMTs) are chemical labels used for the quantification and identification of proteins, peptides, and other biological macromolecules (Dayon et al., 2008). Tandem MS (MS/MS) using TMTs has become the most powerful and popular proteomics method in recent years (Moulder et al., 2018).

Here, we employed NP methodology to characterize the active component and identify the likely targets and biological pathways of artemisinin. Specifically, the TMT-based quantitative MS/MS proteomic method was applied to identify the various proteins involved in the different activities. Lastly, the core targets of ART were experimentally verified to elucidate the mechanism of its action on BMECs. Based on these results, we selected potential biomarkers that were particularly responsive to artemisinin addition, and obtained new insights on improving the milk synthesis capacity of bovine mammary gland.

## 2 Materials and methods

### 2.1 Screening for active components and predicting targets of artemisinin

For this investigation, the potential active compounds were identified using the TCM pharmacology database and analytical platform (TCMSP, https://old.tcmsp-e.com/tcmsp.php) (Ru et al., 2014) and corresponding pathways for ADME (absorption, distribution, metabolism, and excretion). The components with oral bioavailability (OB) of ≥30% (Xu et al., 2012) and drug-likeness (DL) of≥ 0.18 (Tao et al., 2013) were selected for further analysis. Screening according to the identified compounds, their potential targets were obtained using Swiss Target Prediction tools (http://www.swisstargetprediction.ch) (Daina et al., 2019).

### 2.2 Selection of target proteins related to mammary glands and milk

Genecards (https://www.genecards.org/), which is a human genomic database that automatically integrates sources from 150 websites (Marilyn et al., 2010), was used to select related genes by setting the search words either as “mammary gland” or “milk”. The universal Protein database (UniProt, https://www. UniProt.org/) took the aforementioned data sets and target information and converted the protein names to the corresponding gene names. Lastly, we acquired all “mammary gland” and “milk” hits after deleting repetitions.

### 2.3 Building an interactive network of compounds, targets, and pathways

The selected ART-related targets and mammary gland and milk-related hits were organized into a Venn diagram (https://bioinfogp.cnb.csic.es/tools/venny/) to show intersecting targets with their corresponding active components. To delineate possible mechanisms for ART’s effects on milk biosynthesis, functional information was obtained and analyzed using the database for annotation, visualization, and integrated discovery (DAVID, ver 6.8, https://David.ncifcrf.gov/); *p* < 0.05 was set as the criterion for selecting significantly different values. A visual representation of the common target network compounds for mammary gland/milk data was developed using Cytoscape 3.8.2 (http://www.cytoscape.org/) to reflect their complex interactions with ART. Cytoscape 3.8.2 is open-source software useful to illustrate multicomponent molecular networks and integrate various types of data. Within the network, the nodes represent the ART compounds and their mammary gland- and milk-related targets. The edges show the relationships between the nodes: the quantity of each edge is defined as “degree”.

### 2.4 Cell culture and treatment

Artemisinin (white power, 98% purity) was purchased from Sigma Aldrich (cat # 36159, CAS 63968–64–9) (Shanghai, China).

The bovine mammary epithelial cells (BMECs) used in this study (Mengmeng et al., 2019) were a gift of the Laboratory of Animal Biochemistry and Molecular Biology, Northeast Agricultural University. The BMECs, separated, purified, and identified according to a previous report (Tong et al., 2012) were seeded in six-well plates (Corning, United States) at 10^6^ cells/well and cultured in Dulbecco’s modified Eagle’s medium: nutrient mixture F12 (DMEM-F12, Gibco, United States) containing 10% Australian fetal bovine serum (FBS, Gibco, United States) and 1% penicillin-streptomycin (Gibco, United States) in a humidified incubator with 5% CO_2_ at 37°C. Cell viability was measured by MTT assay after incubation with different concentrations of artemisinin for various durations as described in our previous study (Hou Kun et al., 2021). The optimal effect on cell viability was observed at a concentration of 60 μM artemisinin and a treatment duration of 12 h, and these conditions were used in all subsequent experiments. All experiments were performed in triplicate. The experiments were approved by the Animal Ethics Committee of Beijing University of Agriculture (BUAEC 2020–0211).

### 2.5 Protein extraction, digestion, and labeling with TMT reagents

For TMT-based quantitative proteomic analysis, cells were pretreated with 60 μM artemisinin for 12 h. After washing 3 × with cooled phosphate-buffered saline (PBS, Gibco, United States), cells were suspended 1:10 in 800 μL of RIPA lysis buffer plus protease inhibitors and held on ice for 10 min. Lysates were stored at −80°C until processing. Thawed lysates were centrifuged at 30,000 g for 15 min at 4°C, supernatants were transferred to clean tubes and protein concentration was determined by BCA assay (Beyotime Biotechnology, Shanghai, China). Aliquots (20 μg) of each sample were analyzed by SDS-PAGE followed by Coomassie brilliant blue staining (Beyotime Biotechnology, Shanghai, China) to compare the protein expression among the samples. For proteomics analysis, TCEP was added to the samples at a final concentration of 10 mM and incubated at 37°C for 1 h. Next, iodoacetamide was added (40 mM), and samples were incubated in darkness at RT for 40 min. Solutions were then mixed with chilled acetone (1:6) and stored at −20°C for 4 hours. Precipitates were recovered by centrifugation at 10,000 g for 20 min and dissolved in 150 µL of 100 mM TEAB. Trypsin was added to the suspension (1:50, trypsin: substrate) and digested at 37°C overnight.

The resulting peptides were desalted on C18 columns (Strata X, Phenomenex), dried under vacuum, dissolved in 0.5 M TEAB and labeled with TMT (Thermo, United States) according to the manufacturer’s instructions. The TMT reagent was dissolved in acetonitrile and incubated with peptide for 2 hours. Lastly, samples were mixed with hydroxylamine for 15 min to halt the reaction. The labeled samples were mixed in equal amounts, desalted, and vacuum-dried.

### 2.6 LC-MS analysis

Before LC-MS/MS, the TMT-labeled aliquots (100 µL) were prefractionated on an Agilent 300 Extend C18 column (5 μm particle size, 4.6 mm ID) by HPLC to reduce complexity. The combined fractions were dried, lyophilized, and stored at −80°C until LC-MS/MS analysis.

Tryptic peptides were reconstituted, separated on an EASY-nLC 1000 HPLC system connected to a Q Exactive Plus mass spectrometer (Thermo, United States), and analyzed by MS/MS. The applied electrospray voltage was 2.0 kV. Intact peptides were detected in the Orbitrap at a resolution of 70,000 with an m/z full-scan range of 350–1800. Ion fragments were detected in the Orbitrap at 17,500 resolution with a fixed first-mass of 100 m/z. After the survey scan, a data-dependent mode with automatic alteration (one MS scan followed by twenty MS/MS scans) was used for the top twenty precursor ions above a threshold ion count of 5 × 10^4^ with 30 s dynamic exclusion. Automatic gain control was turned on to stop the Orbitrap from overfilling.

### 2.7 Protein database searching and analysis

The MS/MS data were then analyzed using MaxQuant 1.5.2.8 software. Searches against the NCBI *Bos taurus* database were performed with the following parameters: 10 ppm and 0.02 Da mass tolerance for MS and MS/MS, respectively; two missing cleavages permitted in trypsin digest with fixed modification of cys-carbamidomethylation, N-terminal TMT-6plex, and Lys TMT-6plex; with deamidation (NQ) and oxidation (M) as variable modifications. Peptides extraction was performed with high peptide confidence. False-discovery rate (FDR, 1%) was determined by searching the peptide sequence against a decoy database. For quantitative analysis, a protein must have a minimum of one unique peptide match with TMT ratios.

### 2.8 Bioinformatics analysis

Differentially significant expressed proteins (DSEPs) with a fold change >1.2 or <0.83 and *p* < 0.05 were included in the analysis, and annotated proteins were subjected to gene ontology (GO) and Kyoto Encyclopedia of Genes and Genomes (KEGG) screening. UniProt-GOA was employed to generate GO annotations. The KEGG database served to identify enriched pathways. For GO and KEGG enrichment analysis, a two-tailed *t*-test was used to verify significance of differentially-expressed proteins against all identified proteins. A corrected *p* < 0.05 was deemed significant.

### 2.9 Statistical analyses

Data were assessed using SPSS 21 (IBM, Armonk, NY) and are given as mean ± standard deviation of three independent experiments. Differences were deemed significant at *p* < 0.05 and extremely significant at *p* < 0.01.

## 3 Results

### 3.1 Network pharmacology

#### 3.1.1 Permission to reuse and copyright

In this investigation, 22 compounds were found in ART. The list of compounds and their absorption, distribution, metabolism, and excretion parameters are summarized in Table 1. We also collected the genes related to the search term “mammary gland” and “milk” from GeneCards (https://www.genecards.org). We obtained 214 related targets after verification by Uniprot (https://www.uniprot.org) and removal of repetitive targets.

**TABLE 1:** Main active ingredients of *Artemisia annua* L.

Ingredient ID	Ingredient name	OB (%)	DL	Targets number
MOL002235	Eupatin	50.8	0.41	16
MOL000354	Isorhamnetin	49.6	0.31	60
MOL000359	Sitosterol	36.91	0.75	3
MOL004083	Tamarixetin	32.86	0.31	15
MOL004112	Patuletin	53.11	0.34	11
MOL000422	Kaempferol	41.88	0.24	63
MOL000449	Stigmasterol	43.83	0.76	31
MOL004609	Areapillin	48.96	0.41	17
MOL005229	Artemetin	49.55	0.48	23
MOL000006	Luteolin	36.16	0.25	57
MOL007274	Skrofulein	30.35	0.3	11
MOL007389	Artemisitene	54.36	0.31	-
MOL007400	Vicenin-2_qt	45.84	0.21	-
MOL007401	Cirsiliol	43.46	0.34	10
MOL007404	Vitexin_qt	52.18	0.21	15
MOL007412	DMQT	42.6	0.37	10
MOL007415	[(2S)-2-[[(2S)-2-(benzoylamino)-3-phenylpropanoyl] amino]-3-phenylpropyl] acetate	58.02	0.52	5
MOL007423	6,8-di-c-glucosylapigenin_qt	59.85	0.21	16
MOL007424	Artemisinin	49.88	0.31	25
MOL007425	Dihydroartemisinin	50.75	0.3	-
MOL007426	Deoxyartemisinin	54.47	0.26	1
MOL000098	Quercetin	46.43	0.28	199

#### 3.1.2 Gene ontology and kyoto encyclopedia of genes and genomes analysis of the intersection between artemisinin predicted targets and milk biosynthesis related genes

**FIGURE 1:**
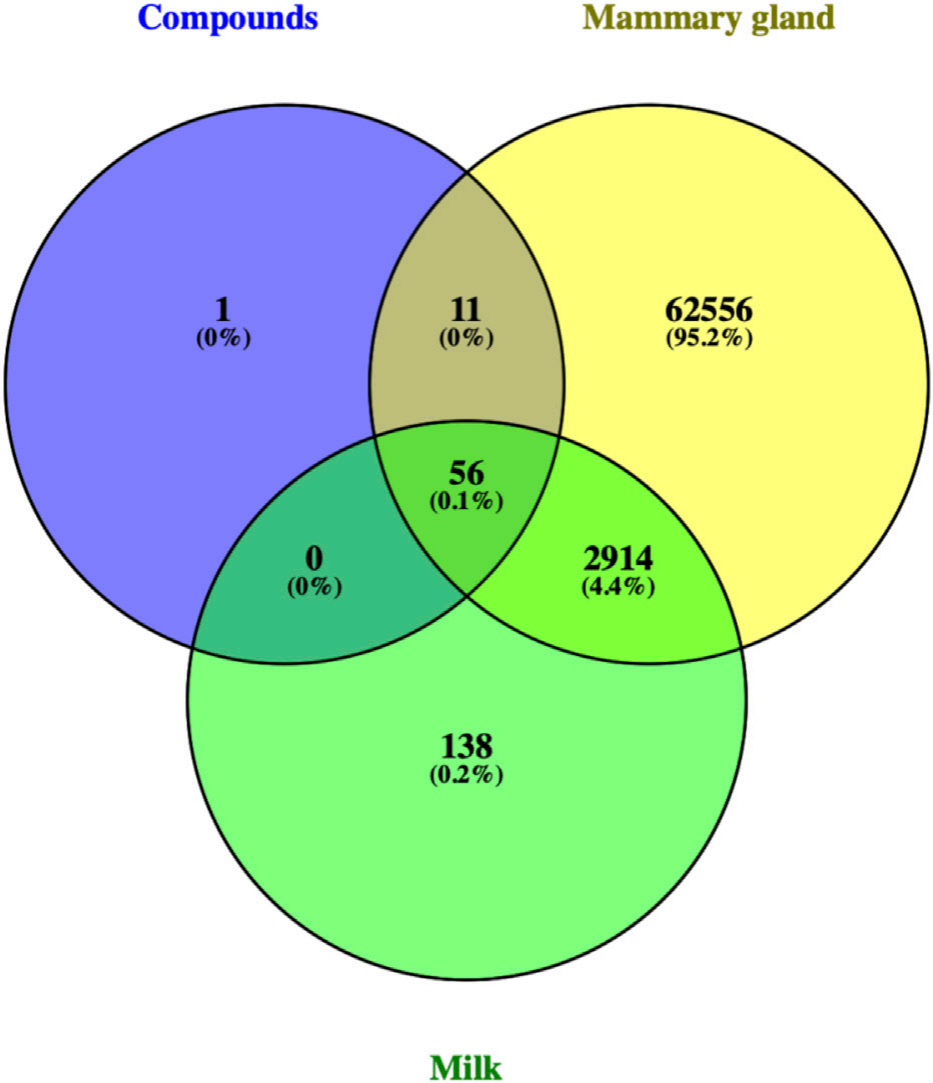
Venn diagram showing intersection of 56 ART-related milk biosynthesis targets.

**FIGURE 2:**
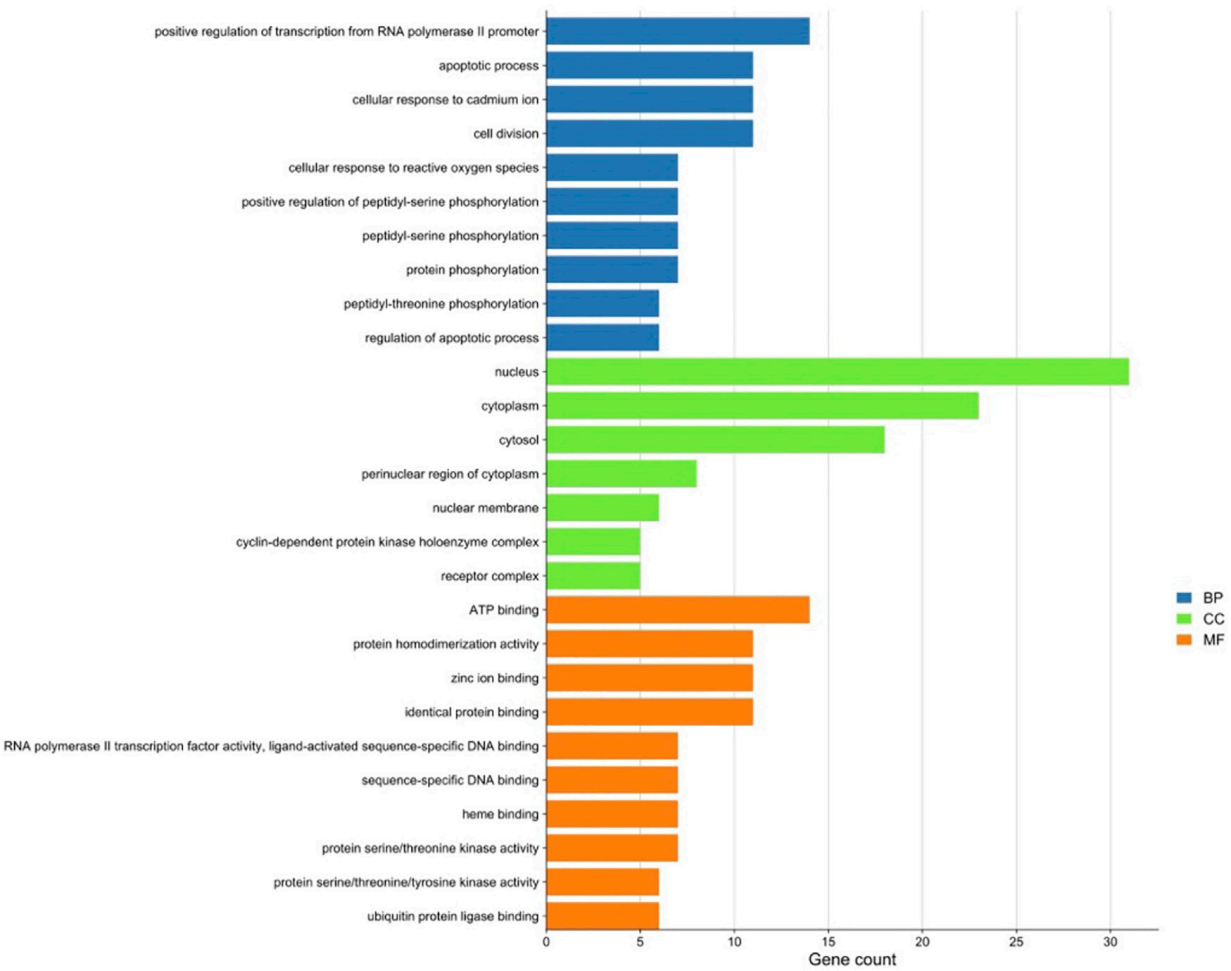
Gene ontology (GO) enrichment analysis of intersected targets. Top ten biological process (BP) terms, cellular component (CC) terms, and molecular function (MF) terms are shown as green, orange, and purple bars, respectively. The *X*-axis represents the gene count of the target, and *Y*-axis represents the GO category of the target gene.

**FIGURE 3:**
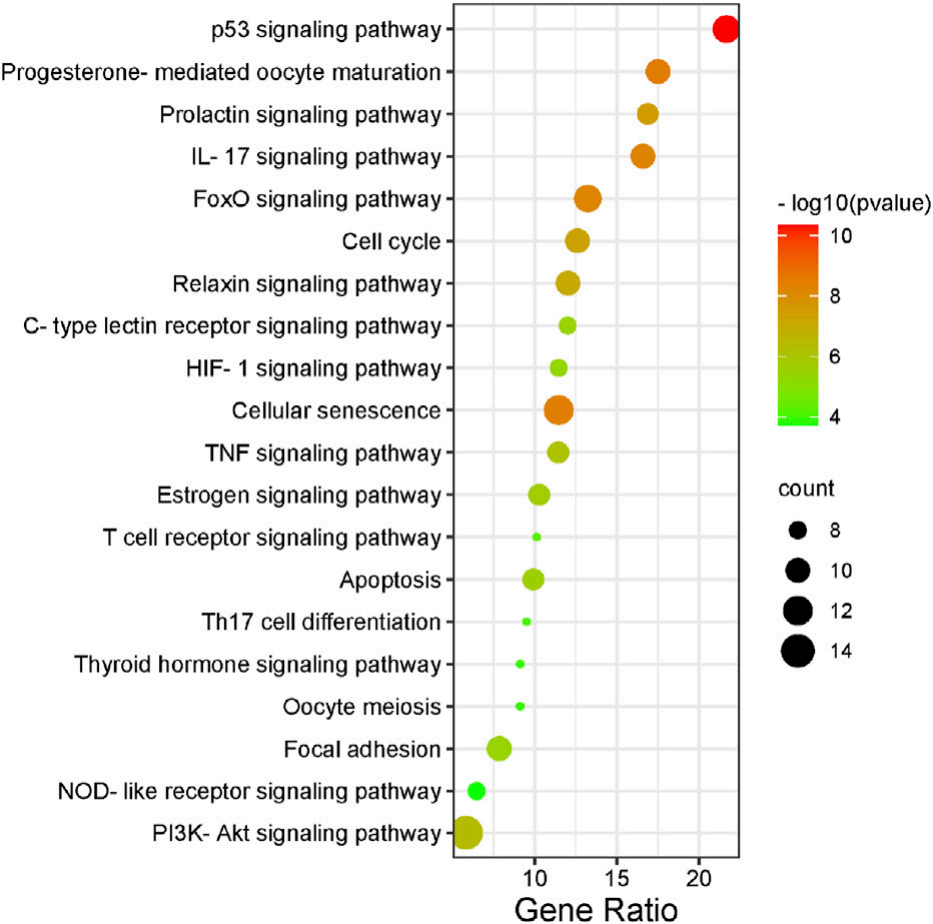
KEGG enrichment analysis of intersected targets. The top 20 KEGG pathways with adjusted *p* < 0.05 were selected and presented in a bubble chart manner. The size of the bubble represents the number of targets enriched in the indicated pathway and the color of the bubble represents the *p* value of enrichment.

There were 56 targets in common between the active compounds and milk biosynthesis-related pathways as shown in Figure 1. All the main targets were then subjected to GO enrichment analysis and KEGG classification. The GO enrichment analysis (Figure 2) was predominantly centred on positive of transcriptional regulation from RNA Pol II promoters, apoptotic processes, cell division, nucleus, cytoplasm, cytosol, ATP-binding, protein homodimerization, and zinc ion binding. KEGG enrichment analysis (Figure 3) indicated pathways related to cellular processes and organismal systems along with specific signalling cascades were included in the most-enriched pathways: p53 signalling, progesterone-mediated oocyte maturation, prolactin signalling, and the PI3K-Akt-related pathway.

#### 3.1.3 Gene ontology and kyoto Encyclopedia of genes and genomes analysis of the intersection between artemisinin predicted targets and milk biosynthesis related genes

**FIGURE 4:**
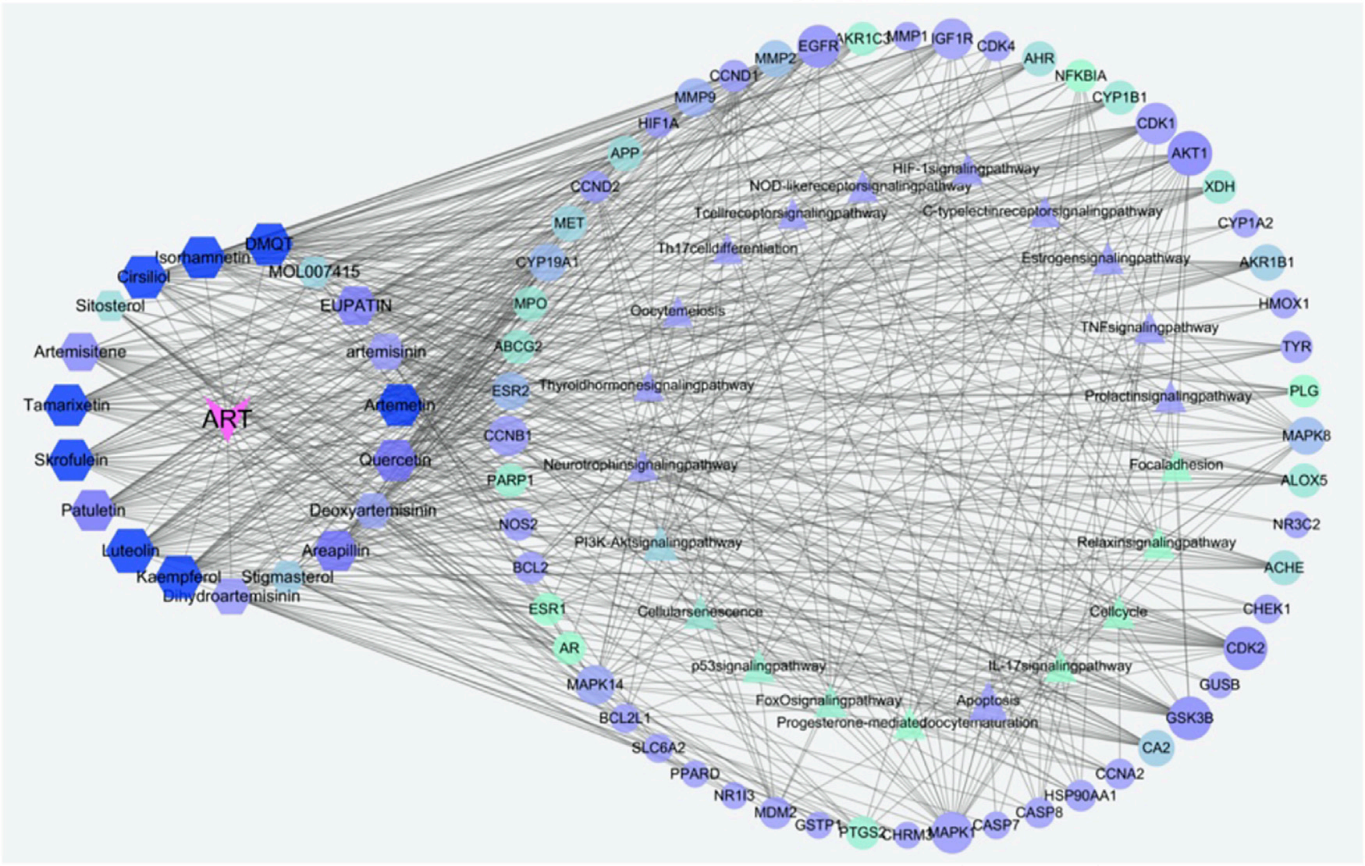
Construction of the compounds-targets-pathways network. The pink V’s represent artemisinin; the hexagons indicate active compounds, the triangular nodes represent KEGG pathways, the elliptical nodes indicate intersected targets. Lines in the figure represent the interaction between two nodes. Both node size and color are in ascending order according to degree.

As an in-depth feature, an integrated visualized network was generated using Cytoscape 3.8.2. The network contained 131 nodes and 720 edges; the more connections there are, the more important the nodes are in the network. The identified active compounds from *Artemisia annua* L could target multiple proteins and trigger complex signalling cascades involved in regulating apoptosis, prolactin and TNF pathways (Figure 4).

### 3.2 Proteomics analysis

#### 3.2.1 Identification of differentially significant expressed proteins in bovine mammary epithelial cells after artemisinin treatment

**FIGURE 5:**
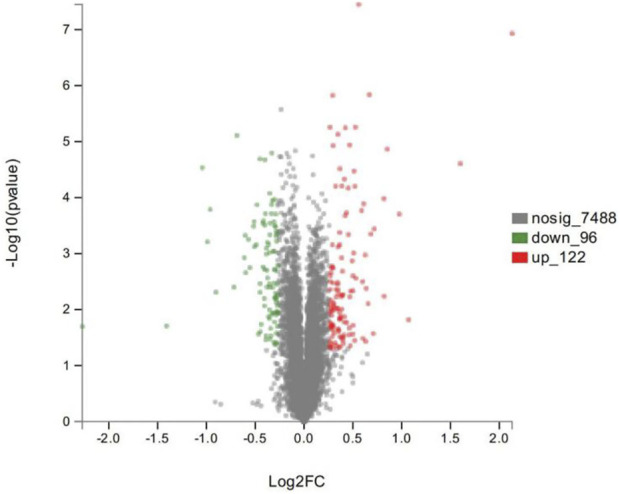
Volcano plot showing significantly up- (red) or down-regulated (green) proteins between the artemisinin treatment group and the control group. The gray points are proteins whose expression levels did not change significantly. The abscissa shows multiples of difference (logarithmic transformation of 2), while the ordinate is significant difference (*p* value, logarithmic transformation of 10).

As shown in the volcano plots in Figure 5, proteins exhibiting fold-change (FC) > 1.20 or <0.83 and *p* < 0.05 in the artemisinin group compared to the control group were regarded as DSEPs. Based on this criterion, 218 DSEPs were recorded, with 122 being significantly upregulated (*red*) and 96 downregulated (*green*) in the artemisinin treatment group relative to control. The top 15 DSEPs (up- and downregulated) are presented in Tables 2, 3. Ribonucleotide reductase subunit 2 (RRM2), G protein-coupled receptor kinase 5 (GRK5), 4F2 cell-surface antigen heavy chain (SLC3A2), wingless-type MMTV integration-site family member 5B (WNT5B), phosphoribosyl pyrophosphate synthase isoform 2 (PPRS2), retinoblastoma (RB1), and choline/ethanolamine phosphotransferase 1 (CEPT1) showed notable upregulation. Downregulated proteins included ferritin heavy chain (FTH1), induced myeloid leukemia cell differentiation protein Mcl-1 (MCL1), HLA class II histocompatibility antigen gamma chain isoform (CD74), ribosomal protein L34 (RPL34), ubiquinone biosynthesis methyltransferase COQ5, mitochondrial precursor (COQ5) and ubiquinone biosynthesis protein COQ7 homolog isoform 1 (COQ7).

**TABLE 2:** Selected significantly upregulated proteins in the artemisinin (ART) group compared with the control (CON) group.

Protein	Description	NCBI accession	FC^a^ (ART/CON) <	Log2FC (ART/CON) <	*p* Value (ART/CON) <
RRM2	Ribonucleotide reductase subunit 2	NP_001231110.1	1.277	0.353	0.005
GRK5	G protein-coupled receptor kinase 5	DAA14699.1	1.322	0.402	0.040
DDIT3	DNA damage-inducible transcript 3 protein	XP_024848055.1	1.965	0.975	< 0.001
SLC3A2	4F2 cell surface antigen heavy chain, member 2	NP_001019659.2	1.225	0.293	< 0.001
WNT5B	Wingless-type MMTV integration site family, member 5B	DAA29179.1	1.205	0.270	0.011
VTN	Vitronectin precursor	NP_001030222.1	1.309	0.389	0.010
RB1	Retinoblastoma 1	DAA23947.1	1.228	0.297	0.020
SLC2A1	Solute carrier family 2, facilitated glucose transporter member 1	DAA30954.1	1.229	0.298	< 0.001
INHBE	Activin beta E-like	DAA29663.1	1.763	0.818	< 0.001
HES-1	Transcription factor HES-1	NP_001029850.1	1.444	0.530	0.003
PRPS2	Phosphoribosyl pyrophosphate synthases isoform 2 <	NP_001107624.1	1.272	0.347	0.022
HSPA5	Endoplasmic reticulum chaperone BiP precursor <	NP_001068616.1	1.251	0.323	< 0.001
ENTPD4	Ectonucleoside triphosphate diphosphohydrolase 4	XP_024851554.1	1.217	0.284	0.013
C17ORF48	Chromosome 17 open reading frame 48	DAA18733.1	1.256	0.329	0.006
CEPT1	Choline/ethanolamine phosphotransferase 1	DAA31515.1	1.240	0.310	0.026

a FC (Fold Change) refers to the multiple of the difference in the expression of the same protein between two samples; FC > 1.2 indicates upregulated proteins, and FC < 0.83 indicates downregulated proteins.

**TABLE 3:** Selected significantly downregulated proteins in the artemisinin (ART) group compared with the control (CON) group.

Protein	Description	NCBI accession	FC (ART/CON) <	Log2FC (ART/CON) <	*p*-value (ART/CON) <
GABARAPL2	Gamma-aminobutyric acid receptor-associated protein-like 2	NP_777100.1	0.786	−0.348	< 0.001
BOLA-DRA	Major histocompatibility complex, class II, DR alpha	DAA16455.1	0.756	−0.403	< 0.001
PLPP3	Phospholipid phosphatase 3	NP_001069941.1	0.766	−0.384	< 0.001
FZD6	Frizzled-6	XP_003586952.2	0.818	−0.289	< 0.001
MCL1	Induced myeloid leukemia cell differentiation protein Mcl-1	NP_001092676.1	0.795	−0.331	< 0.001
GABRP	Gamma-aminobutyric acid receptor subunit pi precursor	NP_001015618.1	0.828	−0.271	0.005
FTH1	Ferritin heavy chain	DAA13737.1	0.824	−0.280	< 0.001
NSD2	Histone-lysine N-methyltransferase NSD2	XP_024849518.1	0.762	−0.392	0.034
UQCRB	Cytochrome b-c1 complex subunit 7	NP_001029969.1	0.812	−0.301	0.002
CD74	HLA class II histocompatibility antigen gamma chain	XP_005209667.1	0.702	−0.509	< 0.001
RPS21	28S ribosomal protein S21	XP_005203988.1	0.750	−0.415	< 0.001
RPL34	Ribosomal protein L34	DAA28201.1	0.485345	−1.043	< 0.001
SORT1	Sortilin 1	DAA31548.1	0.816	−0.293	0.006
COQ5	Ubiquinone biosynthesis methyltransferase COQ5, mitochondrial precursor	DAA20640.1	0.817	−0.292	0.041
COQ7	Ubiquinone biosynthesis protein COQ7 homolog isoform 1	DAA15506.1	0.782	−0.355	0.003

#### 3.2.2 Gene ontology functional enrichment analyses of differentially significant expressed proteins

**FIGURE 6:**
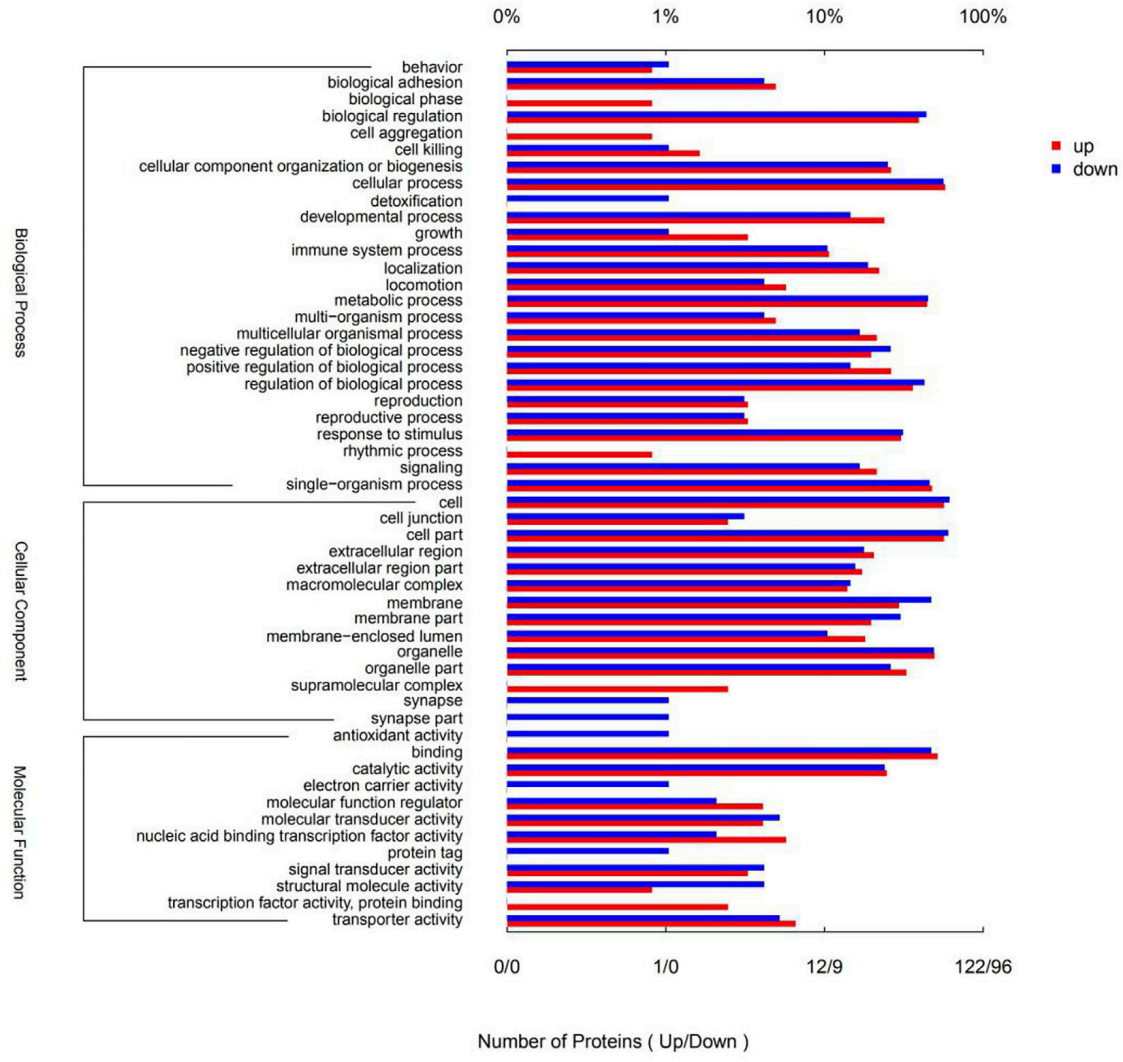
GO terms of the DSEPs in the artemisinin treatment group compared with the control group. The red bars represent up-regulated proteins, and the blue bars represent down-regulated proteins.

Gene ontology (GO) is a conventional gene-function classification system yielding a list of dynamically updated, standardized functionality terms that enable functional interpretation of DSEPs. The 218 DSEPs identified here are classed in 52 GO terms: 26 biological processes (BP), 14 cellular components (CC), and 12 molecular functions (MF) (Figure 6). All terms except detoxification, synapses, antioxidant activity, electron carrier activity and protein tags were represented by upregulated proteins. By contrast, all terms except biological phase, cell aggregation, rhythmic processes, supramolecular complexes, transcription factor activity and protein binding were represented by downregulated proteins. In BP ontology, the DSEPs were mainly related to cellular processes (70 up- and 54 downregulated proteins), metabolic processes (54 up- and 43 downregulated proteins), biological regulation (48 up- and 42 downregulated proteins), regulation of biological processes (44 up- and 41 downregulated proteins), response to stimuli (37 up- and 30 downregulated proteins), cellular component organization or biogenesis (32 up- and 24 downregulated proteins) and developmental processes (29 up- and 14 downregulated proteins). In the CC ontology, the DSEPs were mainly associated with cell, cell part, organelle, membrane, organelle part and membrane part terms. In the GO MF annotation category, the DSEPs were mainly involved in binding (63 up- and 45 downregulated proteins) and catalytic activity (30 up- and 23 downregulated proteins).

**FIGURE 7:**
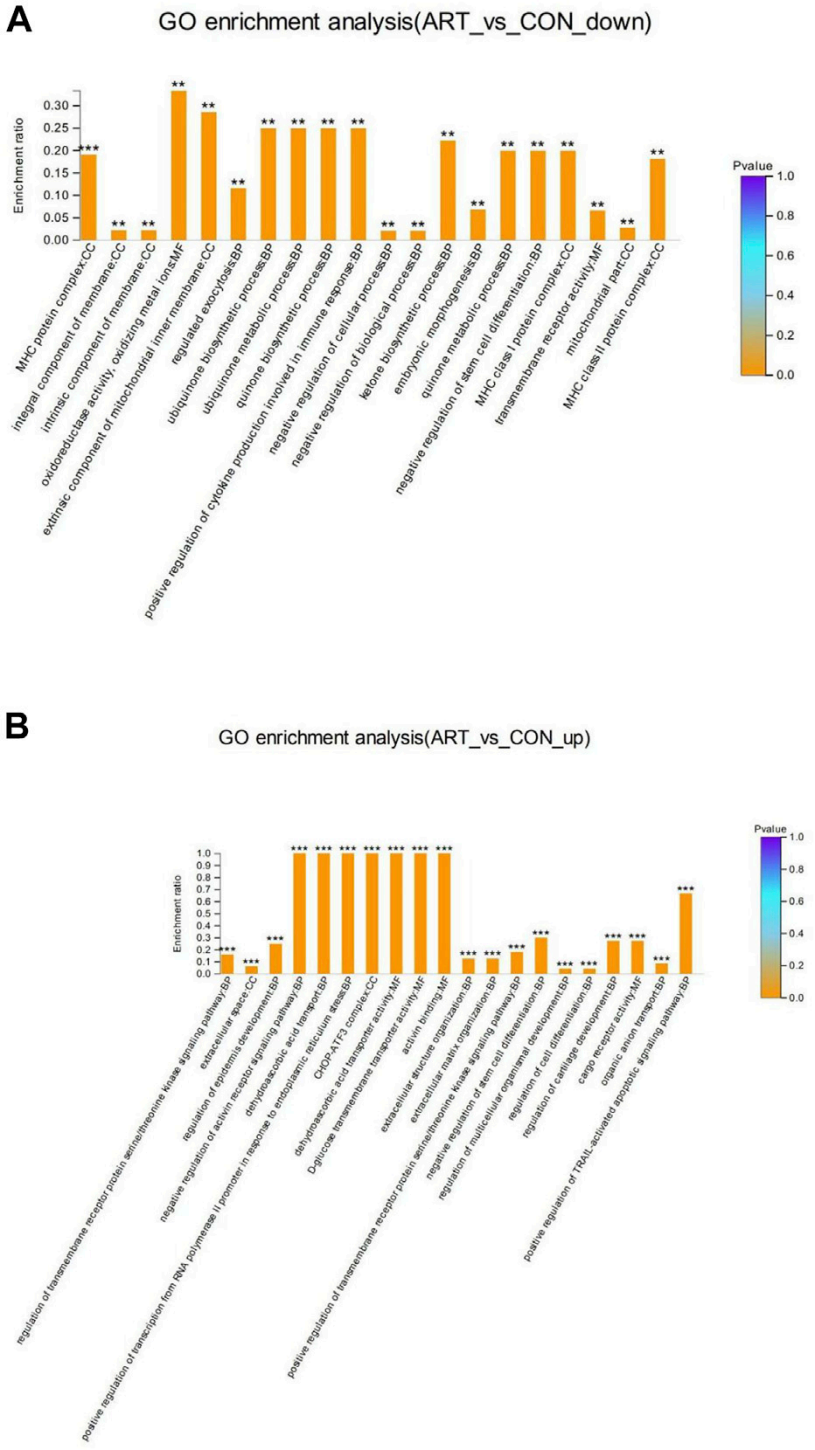
GO enrichment analysis of DSEPs in the artemisinin treatment group vs. the control group. **(A)** GO enrichment analysis of upregulated DSEPs. **(B)** GO enrichment analysis of downregulated DSEPs. The *x*-axis represents the GO terms, while the *y*-axis represents the enrichment ratio, which is the ratio of the number of DSEPs in the GO term to the number of all annotated proteins in the GO term. The greater the ratio, the greater the degree of enrichment in each category. The color of the bars is related to the *p* value: ***p < 0.001, **p < 0.01, and*p < 0.05.

GO enrichment analysis of the DSEPs was performed to clarify the differences at the functional level. Figure 7A displays the GO enrichment results for the up-regulated DSEPs in the artemisinin group versus the control group. The significantly enriched GO terms belonging to BP included negative regulation of the activin-receptor signal pathway, extracellular structure organization, control of cell differentiation, and organic anion transport. Among the CC terms, the CHOP-ATF3 complex was significantly enriched. In the MF group, terms such as dehydroascorbic acid transporter activity, d-glucose transmembrane transporter activity, and activin binding were significantly enriched. These results suggest that treatment of BMECs with artemisinin resulted in greater enrichment in the induction of apoptosis and promotion of differentiation.

The results of GO enrichment analysis of the downregulated DSEPs in the artemisinin group compared to control are shown in Figure 7B. Many GO terms were strongly enriched, including the biosynthetic processes for ubiquinone, quinones and ketones, the metabolic processes of ubiquinone and quinones, and the positive regulation of cytokine production involved in the immune response in BP ontology; MHC protein complexes, MHC class I protein complexes, MHC class II protein complexes and extrinsic components of the mitochondrial inner membrane in the CC ontology; and oxidoreductase activity and oxidizing metal ions in the MF ontology. These results indicate that artemisinin treatment improves antioxidant capacity and immunity of BMECs, which would aid in the normal development of mammary gland function in dairy cows.

### 3.3 Kyoto encyclopedia of genes and genomes pathway enrichment analysis

**FIGURE 8:**
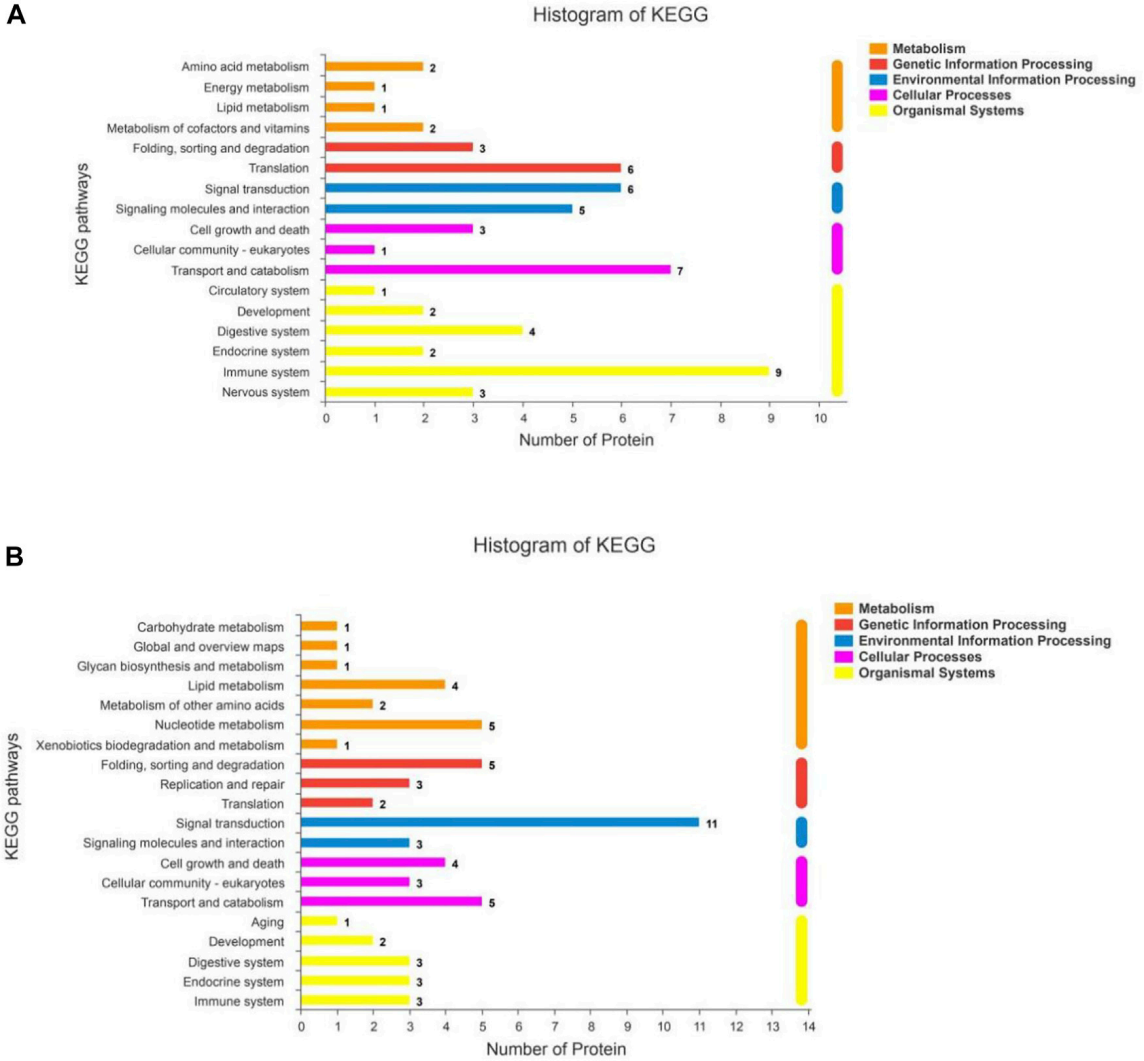
KEGG pathway analysis for DSEPs in the artemisinin group compared to the control group. **(A)** Upregulated DSEPs and **(B)** downregulated DSEPs. The ordinate is the name of the KEGG metabolic pathway, and the abscissa is the number of proteins annotated to the pathway. The bars are color-coded according to KEGG pathways, and the length of each bar corresponds to the number of proteins annotated to the corresponding pathway.

The KEGG statistics from the enrichment analyses of the up- and downregulated DSEPs are provided in Figures 8A,B, respectively. The upregulated DSEPs were assigned to 20 pathways: seven metabolic pathways, three genetic information-processing pathways, two environmental information-processing pathways, three cellular process pathways, and five organismal systems pathways. The up-regulated DSEPs were mainly enriched in lipid metabolism, nucleotide metabolism, folding, sorting and degradation, signal transduction, cell growth and death, transport and catabolism, and substance dependence. The downregulated DSEPs were mainly enriched in 17 pathways, including six organismal systems pathways, four metabolic pathways, two genetic information-processing pathways, two environmental information-processing pathways, and three cellular process pathways. The main enriched pathways were translation, signal transduction, signaling molecules and interactions, transport and catabolism, and immunoresponses. These significantly enriched pathways play important roles in improving the proliferation and differentiation of BMECs, promoting mammary gland development, and regulating metabolism related to milk biosynthesis.

**FIGURE 9:**
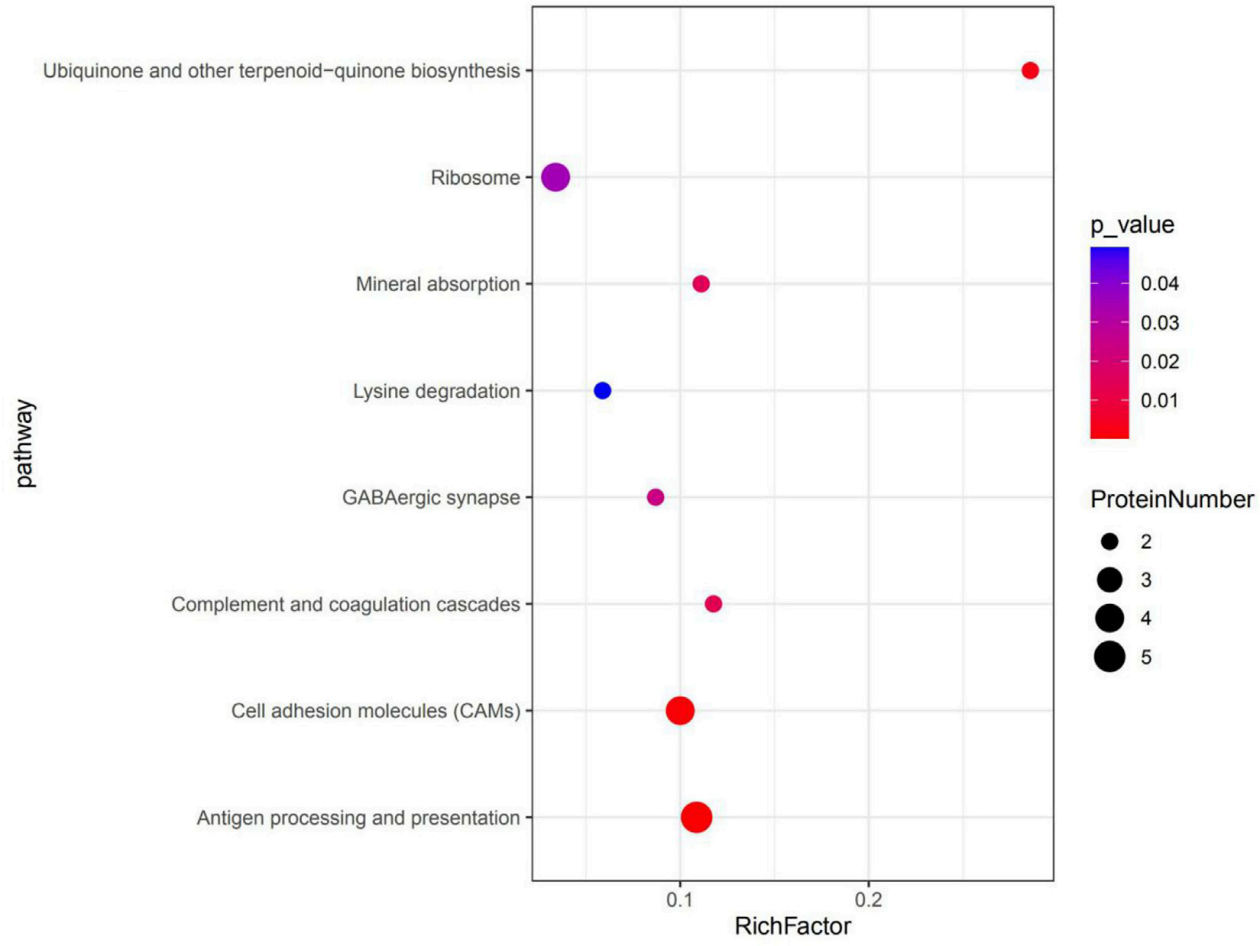
The top eight KEGG pathways enriched in DSEPs. The *X*-axis represents the corresponding pathway enrichment score; the *Y*-axis represents the name of each pathway. The enrichment score is the ratio of the number of DSEPs in the pathway to the number of all annotated proteins in the pathway; a higher enrichment score indicates a higher degree of enrichment, and a larger diameter of the circle indicates a greater number of DSEPs.

KEGG pathway enrichment analyses were used to determine the potential functions of these DSEPs by classifying the DSEPs of artemisinin-treated BMECs into the eight most highly enriched pathways (Figure 9). Among immunomodulatory activities, the most highly enriched pathways were associated with mineral absorption, the GABAergic synapse, complement and coagulatory cascades, and antigen processing and presentation. The corresponding DSEPs included GABARAPL2, FTH1, BOLA-DRA and GABPR. Two pathways related to metabolism, the ubiquinone and other terpenoid-quinone biosynthesis and lysine degradation were overrepresented, and the corresponding DSEPs included COQ5, COQ7 and NSD2. One pathway related to environmental information processing involving cell adhesion molecules (CAMs) was overrepresented, and the corresponding DSEPs and BOLA-DRA had the lowest *p*-values among the identified pathways. Lastly, one pathway related to genetic information processing, the ribosome pathway, was overrepresented. The ribosome pathway was the most significant down-regulated pathway, and the corresponding DSEP was RPL34. These results suggest that artemisinin treatment can enhance BMEC proliferation and mammary gland development.

## 4 Discussion

Artemisinin is a 1, 2, -trioxane isolated from the Chinese medicinal plant, sweet wormwood (*Artemisia annua* L., Asteraceae) (Efferth, 2017), which has antimalarial and antiparasitic properties and is widely used in animal husbandry. In addition, artemisinin has antibacterial, anti-inflammatory and antioxidant effects and promotes cell proliferation and differentiation. In our previous study, artemisinin significantly improved milk quality in mid-lactation dairy cows, increased milk production, and antioxidant capacity (Hou Kun, 2019; Hou et al., 2020). However, because of the multiple components of artemisinin, it was difficult to identify all the active substances and describe their mechanism of action in mammary gland regulation and milk biosynthesis in BMECs. Thus, we employed systematic methods involving network pharmacology and proteomics to determine the molecular mechanisms, underlying the effects of artemisinin on BMECs. The results of our investigation should allow the development of a dietary approach for the application of artemisinin in improving milk production on dairy farms.

Our work resulted in the identification of 22 active components from artemisinin. Among these were quercetin, areapillin, eupatin and patuletin, which have been shown to be active in mammary gland development. Previous studies have proved that the common plant bioflavonoid, quercetin, has a variety of pharmacological activities, including anti-inflammatory, antioxidant, anticancer, antiviral, and antibacterial effects. Areapillin was reported to possess anti-inflammatory, antibacterial, and immunostimulatory activity (Kłósek et al., 2021). Eupatin showed marked inhibitory effects on neuroinflammation and tau phosphorylation, which supported the conclusion that it has significant antiinflammatory properties (Chou et al., 2020). A previous study demonstrated that patuletin was able to inhibit FASN and showed antiproliferative and pro-apoptotic activities against human breast cancer cells (Zhu et al., 2017). These studies support the hypothesis that artemisinin is important in mammary gland development through the synergistic effects of its bioactive compounds.

In the present study, a proteomics approach based on TMT was used to identify DSEPs in BMECs treated with artemisinin. Our results suggested that the DSEPs play essential roles in the molecular mechanism of the effects of artemisinin on BMECs and are involved in biological processes, cellular components, and molecular functions. The results of GO enrichment screening of the DSEPs demonstrated their potential physiological involvement in promoting bovine mammary gland development and BMEC proliferation, differentiation, and lipid synthesis. In the present study, proteomics analysis showed that artemisinin significantly upregulated RRM2 in BMECs. Previous studied showed that RRM2 is a rate-limiting enzyme for DNA synthesis/repair during S-phase, and its expression is cell-cycle dependent (Wang et al., 2018). Specifically, RRM2 expression occurs during late G1/early S-phase and the protein undergoes degradation in late S-phase (Engstrm et al., 1985). Regulating cell-cycle progression is a key factor in inhibiting cell proliferation (Vermeulen et al., 2010). In cancer cells it was demonstrated that downregulation of RRM2 significantly induced apoptosis and prevented cell-cycle progression at G1 (Wang et al., 2018). Thus, we speculate that artemisinin may modulate the cell cycle by up-regulating RRM2 to promote cell proliferation, but the mechanism needs further in-depth study.

GO enrichment analysis revealed that DSEPs upregulated by artemisinin treatment were primarily associated with negative regulation of the activin receptor signaling pathway, upregulation of RNA polymerase II transcription as a result of ER stress, d-glucose transmembrane transporter activity, activin binding and formation of the CHOP-ATF3 complex. Artemisinin also up-regulated SLC3A2 (amino acid transporter heavy chain, member 2), a transmembrane cell-surface protein of the solute-carrier family that is also an ER stress-induced protein. The Ca^2+^-ATPase inhibitor, thapsigargin (TG), induces apoptosis, and flow cytometry analysis has shown that SLC3A2 inhibition enhances apoptosis induced by TG (Liu et al., 2018). Our results indicate that SLC3A2 may be instrumental in protecting injured cells from apoptosis, as the SLC3A2 up-regulation observed here may protect cells from apoptosis. However, elucidating the SLC3A2 function fully will need further work. RB1, a down-stream target of cyclin-dependent kinase (CDK) 4/6 inhibitors (Knudsen et al., 2019), was upregulated by artemisinin in the present study, and GO enrichment analysis showed that it could act by regulating cell-cycle progression. Studies in Rb1-knockdown mice have shown that RB1 deficiency not only results in deregulation of proliferation but also causes extensive apoptosis in the nervous system, the lens, and skeletal muscle (Indovina et al., 2015). Thus, we hypothesized that upregulation of RB1 by artemisinin promoted cell proliferation and inhibited apoptosis. Another up-regulated protein, PRPS2, is the rate-limiting enzyme in the biosynthetic pathway of purines (Qiao et al., 2020), which might also play a major role in response to artemisinin in BMECs. It has been reported that PRPS2 is necessary to promote protein synthesis and nucleotide synthesis to maintain cell proliferation. Knockdown of PRPS2 significantly upregulated the cyclin-dependent kinase inhibitor, p27, but downregulated the G1/S protein, cyclin D1, to induce apoptosis and cell-cycle arrest in G1, ultimately inhibiting proliferation (Qiao et al., 2020). The mechanism of artemisinin’s regulation of BMECs needs further research, as it may either upregulate cell proliferation or inhibit apoptosis in BMECs.

Among the proteins that were significantly downregulated by artemisinin treatment, CD74 has been shown to be important for antigen presentation as a mediator of the MHC II complex construction and cell trafficking (Gil-Yarom et al., 2017). Moreover, CD74 has also been implicated in several processes separate from MHC II assembly, such as endosomal trafficking, cell migration and signal transduction, as a receptor of the pro-inflammatory cytokine, macrophage migration inhibitory factor (MIF) (Schrder and, 2016). MIF binds to CD74, inducing intra-membrane cleavage and release of its intracellular cytosolic domain (CD74-ICD), which regulates cell survival. CD74 was demonstrated to be upregulated in various types of cancer and associated with abnormal cell growth and metastasis. Therefore, we speculated that CD74 also influenced the growth and survival of BMECs in response to artemisinin. The ribosomal protein, RPL34, is conserved from archaea to eukaryotes and regulates the growth of prokaryotes, and the cells of plants and animals (Feng et al., 2016). RPL34 expression is significantly upregulated in tumor cells compared to adjacent healthy tissues, and over-expression of RPL34 may promote the abnormal proliferation of cancer cells. RPL34 knockdown by shRNA significantly decreased cell proliferation and increased apoptosis and S-phase arrest (Yang, 2016). Consistent with their findings, our study proved that artemisinin treatment downregulated RPL34, thereby regulating the cell cycle and contributing to apoptosis. Another down-regulated protein, COQ7, is the central regulatory factor of coenzyme Q (COQ) biosynthesis (Lunt and Heiden, 2011). Downregulation of COQ7 leads to a decrease in the rate of COQ biosynthesis and respiratory chain activity in mitochondria, thereby influencing oxidative phosphorylation and the switch to enhanced aerobic glycolysis (Cascajo et al., 2016). As many cells perform aerobic glycolysis during high proliferative rates, COQ7 may be essential in promoting cell growth. Future investigations of these proteins may uncover fresh pathways for deciphering the mechanism of artemisinin’s actions on BMECs.

By contrast, the downregulated DSEPs in BMECs treated with artemisinin as analyzed by GO enrichment were associated mainly with ubiquinone biosynthesis, quinone metabolism, positive regulation of cytokines that are part of the immune system, formation of MHC class I and II complexes, and extrinsic component of the mitochondrial inner membrane. These results were in line with the KEGG enrichment analysis showing strong linkage to ubiquinone and other terpenoid-quinone biosynthesis. It has been reported that ubiquinone (CoQ) is essential for electron transfer through the respiratory chain in mitochondria. Ubiquinone is the primary source of mitochondrial reactive oxygen species (ROS) but also acts as an antioxidant (Wang and Hekimi, 2019). Quinones are highly redox-active molecules, and their generation of semiquinone radical anions in the redox cycle can lead to ROS formation (Bolton and Dunlap, 2017). ROS can cause DNA damage that seriously impacts cellular integrity, leading to perturbation of DNA replication and cell division that ultimately results in cell-cycle arrest and apoptosis (Jie et al., 2018). MHC class I complexes bind peptide fragments derived from protein processing and present them on cell surfaces where they are bound by T lymphocytes through their receptors (TCRs) or by natural killer (NK) cells (Dixon and Syamal, 2018). This recognition is a major part of autoimmunity and the immune response to pathogenic bacteria and viruses, and tumor cells. Therefore, the downregulation of these DSEPs by artemisinin treatment may play a role in immune and antioxidant activities in BMECs.

Antigen presentation by MHC complexes together with costimulatory molecules results in release of proinflammatory cytokines that are part of an effective immune response (Cresswell, 2005). Our results suggest that the most significantly enriched pathways are involved in antigen processing and presentation, CAMs, and the ribosome, which play essential roles in BMEC proliferation. Thus, artemisinin treatment can improve the immune capacity of BMECs and contribute to mammary gland development and function. A previous study proved that transmembrane CAM proteins are involved in cell adhesion and other interactions among cells or between cells and the extracellular matrix (Ling et al., 2012). CAMs were found at pre- and postsynaptic sites where they triggered synaptic differentiation through crosstalk with intra- and extracellular scaffolds (Bukalo and Dityatev, 2012). These reactions are necessary to bring the synaptic transduction machinery together and ensure proper binding of cell-adhesion sites to cytoskeletal proteins. Cytoskeletal remodeling is essential for cell proliferation, adhesion, and migration (Ribeiro et al., 2018). The enrichment of these pathways implies that artemisinin treatment greatly enhanced protein expression and synthesis to meet increased cell growth requirements (Zhong et al., 2019). Overall, these results indicate that artemisinin can improve the proliferation, differentiation, and migration of BMECs, and the significant changes in proteins related to these pathways may indicate that artemisinin can improve mammary gland development.

## 5 Conclusion

In summary, this investigation methodically analyzed the active components, potential targets, and signaling pathways in artemisinin-induced mammary gland development. A visualization of the network of proposed interactions of 19 bioactive compounds was generated, including 57 key targets in mammary gland milk biosynthesis. In addition, 218 DSEP from artemisinin-treated BMECs were identified by a TMT-based proteomics technique. Among the DSEPs, 122 were upregulated, including RRM2, SLC3A2, RB1, and PRPS2, and 96 were downregulated, including CD74, RPL34 and COQ7. GO annotation analysis revealed that the DSEPs were mostly associated with cell proliferation, apoptosis, differentiation, and migration. Specifically, GO enrichment analysis indicated that the upregulated DSEPs may play a role in promoting cell proliferation and regulating cell apoptosis, while the downregulated DSEPs may be involved in the immune and antioxidant activities of BMECs. KEGG pathway analysis indicated enrichment of pathways related to antigen processing and presentation, CAMs, and ribosomes, which play important roles in the normal development of the mammary gland and milk production. The identification of these proteins provides a foundation for further studies of the mechanism by which artemisinin may improve mammary gland biosynthesis in dairy cows.

## Data Availability

The datasets presented in this study can be found in online repositories. The names of the repository/repositories and accession number(s) can be found in the article/supplementary material.

## References

[B1] Bgab E. Cpz A. Cza B. Yao X. Gy D. Liang L. (2021). Elucidation of the anti-inflammatory mechanism of Er miao san by integrative approach of network pharmacology and experimental verification. Pharmacol. Res. 175, 106000. 10.1016/j.phrs.2021.106000 34838694

[B2] Bilia A. R. Santomauro F. Sacco C. Bergonzi M. C. Donato R. (2014). Essential oil of Artemisia annua L.: An extraordinary component with numerous antimicrobial properties. Evid. Based. Complement. Altern. Med. 2014, 159819. 10.1155/2014/159819 PMC3995097 24799936

[B3] Bolton J. L. Dunlap T. L. (2017). formation and biological targets of quinones: Cytotoxic versus cytoprotective effects. Chem. Res. Toxicol. 30 (1), 13–37. 10.1021/acs.chemrestox.6b00256 27617882 PMC5241708

[B4] Bukalo O. Dityatev A. (2012). Synaptic cell adhesion molecules. Adv. Exp. Med. Biol. 970, 97–128. 10.1007/978-3-7091-0932-8_5 22351053

[B5] Cascajo M. V. Abdelmohsen K. Noh J. H. Fernández-Ayala D. J. M. Willers I. M. Brea G. (2016). RNA-binding proteins regulate cell respiration and coenzyme Q biosynthesis by post-transcriptional regulation of COQ7. RNA Biol. 13, 622–634. 10.1080/15476286.2015.1119366 26690054 PMC7609068

[B6] Chou C. H. Hsu K. C. Lin T. E. Yang C. R. (2020). Anti-inflammatory and tau phosphorylation–inhibitory effects of eupatin. Molecules 25 (23), 5652. 10.3390/molecules25235652 PMC7731404 33266202

[B7] Cresswell P. Ackerman A. L. Giodini A. Peaper D. R. Wearsch P. A. (2005). Mechanisms of MHC class I-restricted antigen processing and cross-presentation. Immunol. Rev. 207 (1), 145–157. 10.1111/j.0105-2896.2005.00316.x 16181333

[B8] Daina A. Michielin O. Zoete V. (2019). SwissTargetPrediction: Updated data and new features for efficient prediction of protein targets of small molecules. Nucleic Acids Res. 47, W357–W364. 10.1093/nar/gkz382 31106366 PMC6602486

[B9] Dayon L. Hainard A. Licker V. Turck N. Sanchez J. C. Hochstrasser D. F. (2008). Relative quantification of proteins in human cerebrospinal fluids by MS/MS using 6-plex isobaric tags. Anal. Chem. 80 (8), 2921–2931. 10.1021/ac702422x 18312001

[B10] Dixon A. M. Syamal R. (2018). Role of membrane environment and membrane-spanning protein regions in assembly and function of the Class II Major Histocompatibility complex. Hum. Immunol. 80, 5–14. 10.1016/j.humimm.2018.07.004 30102939

[B11] Efferth T. (2017). From ancient herb to versatile, modern drug: Artemisia annua and artemisinin for cancer therapy. Seminars Cancer Biol. 46, 65–83. 10.1016/j.semcancer.2017.02.009 28254675

[B12] Efferth T. (2007). Willmar schwabe award 2006: Antiplasmodial and antitumor activity of artemisinin--from bench to bedside. Planta Med. 73 (4), 299–309. 10.1055/s-2007-967138 17354163

[B13] Engstrm Y. Eriksson S. Jildevik I. Skog S. Tribukait B. (1985). Cell cycle-dependent expression of mammalian ribonucleotide reductase. Differential regulation of the two subunits. J. Biol. Chem. 260 (16), 9114–9116. 10.1016/s0021-9258(17)39337-7 3894352

[B14] Feng W. Ding L. Wei Z. Zhang Y. Yan L. Qinghua L. (2016). Ribosomal protein L34 promotes the proliferation, invasion, and metastasis of pancreatic cancer cells. Oncotarget 7 (51), 85259–85272. 10.18632/oncotarget.13269 27845896 PMC5356734

[B15] Ferreira J. Luthria D. L. Sasaki T. Heyerick A. (2010). Flavonoids from Artemisia annua L. As antioxidants and their potential synergism with artemisinin against malaria and cancer. Molecules 15 (5), 3135–3170. 10.3390/molecules15053135 20657468 PMC6263261

[B16] Gil-Yarom N. Radomir L. Sever L. Kramer M. P. Lewinsky H. Bornstein C. (2017). CD74 is a novel transcription regulator. Proc. Natl. Acad. Sci. U. S. A. 114 (3), 562–567. 10.1073/pnas.1612195114 28031488 PMC5255621

[B17] Hou K. Tong J. Zhang H. Gao S. Jiang L. Niu H. (2020). Microbiome and metabolic changes in milk in response to artemisinin supplementation in dairy cows. Amb. express 10 (154), 154–214. 10.1186/s13568-020-01080-w 32833065 PMC7445214

[B18] Hou Kun L. X. Shen Yiyuan Zhan Jingwei Niu Hui Xiong Benhai Tong Jinjin (2021). Effects of artemisinin on expression of milk fat synthesis related genes in mammary epithelial cells of dairy cows. Anim. Nutr. 33 (11), 6407–6419.

[B19] Hou Kun T. J. Chu Kangkang Xiong Benhai Jiang Linshu (2019). Effects of bamboo leaf flavonoids and Artemisia annua extract on milk performance, milk somatic cell count and serum immune and antioxidant related indexes of dairy cows with subclinical mastitis. Anim. Nutr. 031 (009), 4286–4295.

[B20] Indovina P. Pentimalli F. Casini N. Vocca I. Giordano A. (2015). RB1 dual role in proliferation and apoptosis: Cell fate control and implications for cancer therapy. Oncotarget 6 (20), 17873–17890. 10.18632/oncotarget.4286 26160835 PMC4627222

[B21] Jie M. Tao C. Wu S. Yang C. Zhu Y. Shu K. (2018). iProX: an integrated proteome resource. Nucleic Acids Res. 47, D1211–D1217. 10.1093/nar/gky869 PMC6323926 30252093

[B22] Kłósek M. Sędek Ł. Lewandowska H. Czuba Z. P. (2021). The effect of ethanolic extract of Brazilian green propolis and artepillin C on aFGF-1, Eselectin, and CD40L secreted by human gingival fibroblasts. Cent. Eur. J. Immunol. 46 (4), 438–445. 10.5114/ceji.2021.111215 35125941 PMC8808307

[B23] Knudsen E. S. Pruitt S. C. Hershberger P. A. Witkiewicz A. K. Goodrich D. W. (2019). Cell cycle and beyond: Exploiting new RB1 controlled mechanisms for cancer therapy. Trends Cancer 5 (5), 308–324. 10.1016/j.trecan.2019.03.005 31174843 PMC6719339

[B24] Konkimalla V. B. Blunder M. Korn B. Soomro S. A. Efferth T. Chang W. (2008). Effect of artemisinins and other endoperoxides on nitric oxide-related signaling pathway in RAW 264.7 mouse macrophage cells. Nitric Oxide 19 (2), 184–191. 10.1016/j.niox.2008.04.008 18472018 PMC2582405

[B25] Ling S. Nheu L. Komesaroff P. A. (2012). Cell adhesion molecules as pharmaceutical target in atherosclerosis. Mini Rev. Med. Chem. 12 (2), 175–183. 10.2174/138955712798995057 22070689

[B26] Liu C. Li X. Li C. Zhang Z. Gao X. J. Jia Z. (2018). SLC3A2 is a novel endoplasmic reticulum stress-related signaling protein that regulates the unfolded protein response and apoptosis. PLoS ONE 13 (12), e0208993. 10.1371/journal.pone.0208993 30592731 PMC6310261

[B27] Lunt S. Y. Heiden M. V. (2011). Aerobic glycolysis: Meeting the metabolic requirements of cell proliferation. Annu. Rev. Cell. Dev. Biol. 27 (1), 441–464. 10.1146/annurev-cellbio-092910-154237 21985671

[B28] Marilyn S. Irina D. Justin A. Naomi R. Tsippi I. S. Michael S. (2010). GeneCards version 3: The human gene integrator. Database 2010, baq020. 10.1093/database/baq020 20689021 PMC2938269

[B29] McManaman L. J. (2012). Milk lipid secretion: Recent biomolecular aspects. Biomol. Concepts 3 (6), 581–591. 10.1515/bmc-2012-0025 24605173 PMC3941198

[B30] Mengmeng Y. Luo C. Huang X. Chen D. Li S. Qi H. (2019). Amino acids stimulate glycyl‐tRNA synthetase nuclear localization for mammalian target of rapamycin expression in bovine mammary epithelial cells J. Cell. Physiol. 234 (5), 7608–7621. 10.1002/jcp.27523 30471104

[B31] Moulder R. Bhosale S. D. Goodlett D. R. Lahesmaa R. (2018). Analysis of the plasma proteome using iTRAQ and TMT‐based Isobaric labeling. Mass Spectrom. Rev. 37 (5), 583–606. 10.1002/mas.21550 29120501

[B32] Paddon C. J. Westfall P. J. Pitera D. J. Benjamin K. Fisher K. etal (2013). High-level semi-synthetic production of the potent antimalarial artemisinin. Nature 496 (7446), 528–532. 10.1038/nature12051 23575629

[B33] Qiao H. Tan X. Lv D. J. Xing R. W. Mao X. M. Zhong C. F. (2020). Phosphoribosyl pyrophosphate synthetases 2 knockdown inhibits prostate cancer progression by suppressing cell cycle and inducing cell apoptosis. J. Cancer 11 (5), 1027–1037. 10.7150/jca.37401 31956349 PMC6959080

[B34] Ribeiro L. F. Verpoort B. Wit J. D. (2018). Trafficking mechanisms of synaptogenic cell adhesion molecules. Mol. Cell. Neurosci. 91, 34–47. 10.1016/j.mcn.2018.04.003 29631018

[B35] Ru J. Li P. Wang J. Zhou W. Li B. Huang C. (2014). Tcmsp: A database of systems pharmacology for drug discovery from herbal medicines. J. Cheminform. 6 (1), 13. 10.1186/1758-2946-6-13 24735618 PMC4001360

[B36] Schrder B (2016). The multifaceted roles of the invariant chain CD74 — more than just a chaperone. Biochim. Biophys. Acta 1863 (6), 1269–1281. 10.1016/j.bbamcr.2016.03.026 27033518

[B37] Sheena H. Paul D. Mayumi Y. Catherine D. Nicholas G. (2015). An extract of the medicinal plant Artemisia annua modulates production of inflammatory markers in activated neutrophils. J. Inflamm. Res. 8, 9–14. 10.2147/JIR.S75484 25609991 PMC4298291

[B38] Song X. He Y. Liu M. Yang Y. Mo F. Yan J. (2021). Mechanism underlying polygonum capitatum effect on Helicobacter pylori-associated gastritis based on network pharmacology. Bioorg. Chem. 114 (4), 105044–105137. 10.1016/j.bioorg.2021.105044 34157554

[B39] Tao W. Xue X. Xia W. Li B. Wang Y. Yan L. (2013). Network pharmacology-based prediction of the active ingredients and potential targets of Chinese herbal Radix Curcumae formula for application to cardiovascular disease. J. Ethnopharmacol. 145 (1), 1–10. 10.1016/j.jep.2012.09.051 23142198

[B40] Tian S. Huang P. Gu Y. Yang J. Wu R. Zhao J. (2019). Systems Biology analysis of the effect and mechanism of qi-jing-sheng-Bai granule on leucopenia in mice. Front. Pharmacol. 10 (408), 408–414. 10.3389/fphar.2019.00408 31105563 PMC6494967

[B41] Tong J. J. Ye L. I. Liu R. Gao X. J. Qing-Zhang L. I. (2012). Effect of semen vaccariae and taraxacu mogono on cell adhesion of bovine mammary epithelial cells. J. Integr. Agric. 11 (012), 2043–2050. 10.1016/s2095-3119(12)60462-6

[B42] Turner J. D. Huynh H. T. (1991). Role of tissue remodeling in mammary epithelial cell proliferation and morphogenesis. J. Dairy Sci. 74 (8), 2801–2807. 10.3168/jds.S0022-0302(91)78460-9 1833424

[B43] Vermeulen K. Bockstaele D. R. V. Berneman Z. N. (2010). The cell cycle: A review of regulation, deregulation, and therapeutic targets in cancer. Cell. Prolif. 36 (3), 131–149. 10.1046/j.1365-2184.2003.00266.x PMC6496723 12814430

[B44] Wang N. Li Y. Zhou J. (2018). Downregulation of ribonucleotide reductase subunits M2 induces apoptosis and G1 arrest of cervical cancer cells. Oncol. Lett. 15, 3719–3725. 10.3892/ol.2018.7806 29556274 PMC5844123

[B45] Wilhelm M. Schlegl J. Hahne H. Gholami A. M. Kuster B. Savitski M. M. (2014). Mass-spectrometry-based draft of the human proteome. Nature 509 (7502), 582–587. 10.1038/nature13319 24870543

[B46] Xu X. Zhang W. Huang C. Yan L. Yu H. Wang Y. (2012). A novel chemometric method for the prediction of human oral bioavailability. Int. J. Mol. Sci. 13, 6964–6982. 10.3390/ijms13066964 22837674 PMC3397506

[B47] Yan L. Xiong C. Xu P. Zhu J. Yang Z. Re n H. (2019a). Structural characterization and *in vitro* antitumor activity of A polysaccharide from Artemisia annua L. (Huang Huahao). Carbohydr. Polym. 213, 361–369. 10.1016/j.carbpol.2019.02.081 30879680

[B48] Yan Q. Tang S. Zhou C. Han X. Tan Z. L. (2019b). Effects of free fatty acids with different chain lengths and degrees of saturability on the milk fat synthesis in primary cultured bovine mammary epithelial cells. J. Agric. Food Chem. 67 (31), 8485–8492. 10.1021/acs.jafc.9b02905 31304752

[B49] Yang S. Cui J. Yang Y. Liu Z. Yan H. Tang C. (2016). Over-expressed RPL34 promotes malignant proliferation of non-small cell lung cancer cells. Gene 576, 421–428. 10.1016/j.gene.2015.10.053 26526135

[B50] Wang Y Hekimi S (2019). The complexity of making ubiquinone. Trends Endocrinol. Metab. 30 (12), 929–943. 10.1016/j.tem.2019.08.009 31601461

[B51] Zhang Y. Y. Zhao Z. D. Kong P. Y. Gao L. Yu Y. N. Liu J. (2020). A comparative pharmacogenomic analysis of three classic TCM prescriptions for coronary heart disease based on molecular network modeling. Acta Pharmacol. Sin. 41 (6), 735–744. 10.1038/s41401-019-0352-3 32051552 PMC7471444

[B52] Zhong Q. Wang B. Wang J. Liu Y. Fang X. Liao Z. (2019). Global proteomic analysis of the resuscitation state of Vibrio parahaemolyticus compared with the normal and viable but non-culturable state. Front. Microbiol. 10, 1045. 10.3389/fmicb.2019.01045 31134040 PMC6517545

[B53] Zhu W. Lv C. Wang J. Gao Q. Zhu H. Wen H. (2017). Patuletin induces apoptosis of human breast cancer SK-BR-3 cell line via inhibiting fatty acid synthase gene expression and activity. Oncol. Lett. 14, 7449–7454. 10.3892/ol.2017.7150 29344187 PMC5755210

